# Advances in molecular mechanisms underlying cadmium uptake and translocation in rice

**DOI:** 10.3389/fpls.2022.1003953

**Published:** 2022-09-20

**Authors:** Hao Ai, Daxia Wu, Chunli Li, Mengmeng Hou

**Affiliations:** ^1^Center for Crop Biotechnology, College of Agriculture, Anhui Science and Technology University, Fengyang, China; ^2^State Key Laboratory of Crop Genetics and Germplasm Enhancement, Key Laboratory of Plant Nutrition and Fertilization in Low-Middle Reaches of the Yangtze River, Ministry of Agriculture and Rural Affairs, Nanjing Agricultural University, Nanjing, China; ^3^Shenzhen Branch, Guangdong Laboratory of Lingnan Modern Agriculture, Genome Analysis Laboratory of the Ministry of Agriculture and Rural Affairs, Agricultural Genomics Institute at Shenzhen, Chinese Academy of Agricultural Sciences, Shenzhen, China; ^4^State Key Laboratory of Crop Stress Adaptation and Improvement, School of Life Sciences, Henan University, Kaifeng, China

**Keywords:** cadmium (Cd), translocation, transporter, quantitative trait locus (QTL), rice (*Oryza sativa* L.), uptake

## Abstract

The increasing cadmium (Cd) pollution in paddy fields has severely threatened China’s ecological and food safety. Cultivation of low Cd accumulation varieties to reduce Cd content in rice or cultivation of Cd-tolerant varieties for phytoremediation are considered effective methods to control Cd pollution in paddy fields. However, the underlying molecular mechanism of Cd absorption and transport by rice plants needs to be deciphered to cultivate these varieties. Here, we summarized the molecular mechanisms underlying Cd absorption and transport in rice, as well as the variation of Cd accumulation among rice varieties, the QTLs related to Cd accumulation in rice, and discusses the direction of future research.

## Introduction

Rice is one of the most important food crops grown worldwide. Cadmium (Cd) pollution in rice has attracted great attention from governments around the world. Cd is a notorious heavy metal, which can cause phytotoxicity and human diseases. Thus, effective monitoring of the release of Cd into the environment is imperative. Cd has a strong chemical activity in the soil and is easily absorbed by plants. Cd gets accumulated in the human body through the food chain, thereby severely impacting human health. The rapid increase in Cd pollution in the soil is attributed to the lack of awareness of industrialization and environmental protection in the past three decades. Soils in several parts of China have become more acidic, thus increasing the activity of Cd in soil and its easy uptake by crops (Zhao et al., 2015). Therefore, it is highly essential to understand the absorption and transport mechanism of Cd in rice to reduce its phytotoxicity and human diseases.

## Effects of Cd on human health and plant development

Heavy metal pollution has become a common concern worldwide for agriculture and human health (Bertin and Averbeck, 2006; Mohammed et al., 2011; Clemens et al., 2013; Akesson et al., 2014). Pollution caused by excessive discharge of Cd is prominent; it has been reported that Cd is the third most harmful pollutant to the environment (Jamers et al., 2013) because of its ubiquitous and highly toxic nature (Akesson et al., 2014). Cd can exist in soil for a long time, and its pollution occurs through its irreversible accumulation in the soil (Ismael et al., 2019). In China, nearly 2.786 × 10^5^ ha of farmland have been polluted by Cd, including 5 × 10^4^ t of rice were polluted by Cd, causing serious economic losses (Xu et al., 2014; Liu et al., 2015; Song E. et al., 2015; Li et al., 2017). In addition, Cd pollution is also widespread in Europe (Redondo-Gómez et al., 2010).

Cadmium is produced through industrial activities, including metal mining, zinc refining, and extensive use of herbicides and fertilizers. Once present in the atmosphere, soil, and water, Cd can cause serious problems for all organisms through its bioaccumulation in the food chain (Redondo-Gómez et al., 2010). Contamination of food by Cd primarily occurs *via* contamination of soil and its efficient transfer from soil to plants. Because the biological half-life of Cd is very long (Branca et al., 2020), its content in the body continues to increase with time and eventually settles in the human kidneys. According to a recent Chinese nutrition study, the average Cd intake in China has more than doubled in the 25 years from 1990 to 2015 (Song E. et al., 2017). Therefore, even very low levels of chronic exposure can lead to serious health risks. Cd is the only metal that poses a threat to human and animal health at the plant tissue level—the level that is generally non-phytotoxic (Wang et al., 2011). This implies that plants may not show any toxic symptoms in places with low Cd contamination. However, plants can accumulate Cd in the edible part above allowable levels of human beings. Once these plants enter the food chain, they can result in health problems.

In plants, after Cd enters plant cells, it first acts on mitochondria and chloroplasts and interferes with the electron transport chain (Cannino et al., 2009; Branca et al., 2020). Cd stress can induce the formation of excessive superoxide free radicals and leads to the peroxidation of the cell membrane system (Shah et al., 2001; Haider et al., 2021). Cd is a mutagen that can also inactivate the mismatch repair system in cells (Jin et al., 2003). In addition, Cd can inhibit water transport in plants, resulting in water stress (Haider et al.,2021). It also interferes with the absorption, transport, distribution, and metabolism of essential elements (Rizwan et al., 2016; Haider et al.,2021). With half of the world’s population living on rice, it is an important food crop in the world. When rice grows in the Cd-contaminated soil, excessive Cd accumulates in rice roots, stems, leaves, and grains, which not only hinders the normal growth and development of rice but also seriously affects the quality of rice grain and endangers human health (Uraguchi and Fujiwara, 2012). Therefore, uncovering the uptake and distribution of Cd in plants is essential to help us better understand its accumulation and the tolerance mechanism of rice exposure to Cd.

## Methods of Cd entering plants and factors affecting Cd uptake and translocation in plants

Cadmium is a non-essential element of plants; it enters plants through the absorption channels of essential elements such as calcium (Ca), iron (Fe), manganese (Mn), and zinc (Zn) (Clemens et al., 2006a). Cd can enter root cells either in the form of free ion Cd^2+^ or in the form of chelating compounds such as Cd-phytochelatin (PC) and Cd-glutathione (GSH). The entry of Cd into root cells requires the participation of ion transporters. Several ion transporters exist that can transport Cd^2+^ to root cells (Lux et al., 2011); these ion transporters have element specificity and transport only one or several kinds of metal ions. In addition, there exists a class of ions free of specific cation channel proteins that can transport free Cd^2+^ into cells; these include depolarization-activated calcium channels (DACC), hyperpolarization-activated calcium channels (HACC), and voltage-sensing channels (VICC). These ion channels have no selectivity for cations and thus can transport all cations (Verbruggen et al., 2009; Lux et al., 2011). In addition, Cd can be transported in the form of chelates into root cells by the yellow stripe 1-like (YSL) transporter (Curie et al., 2009).

**Figure 1:**
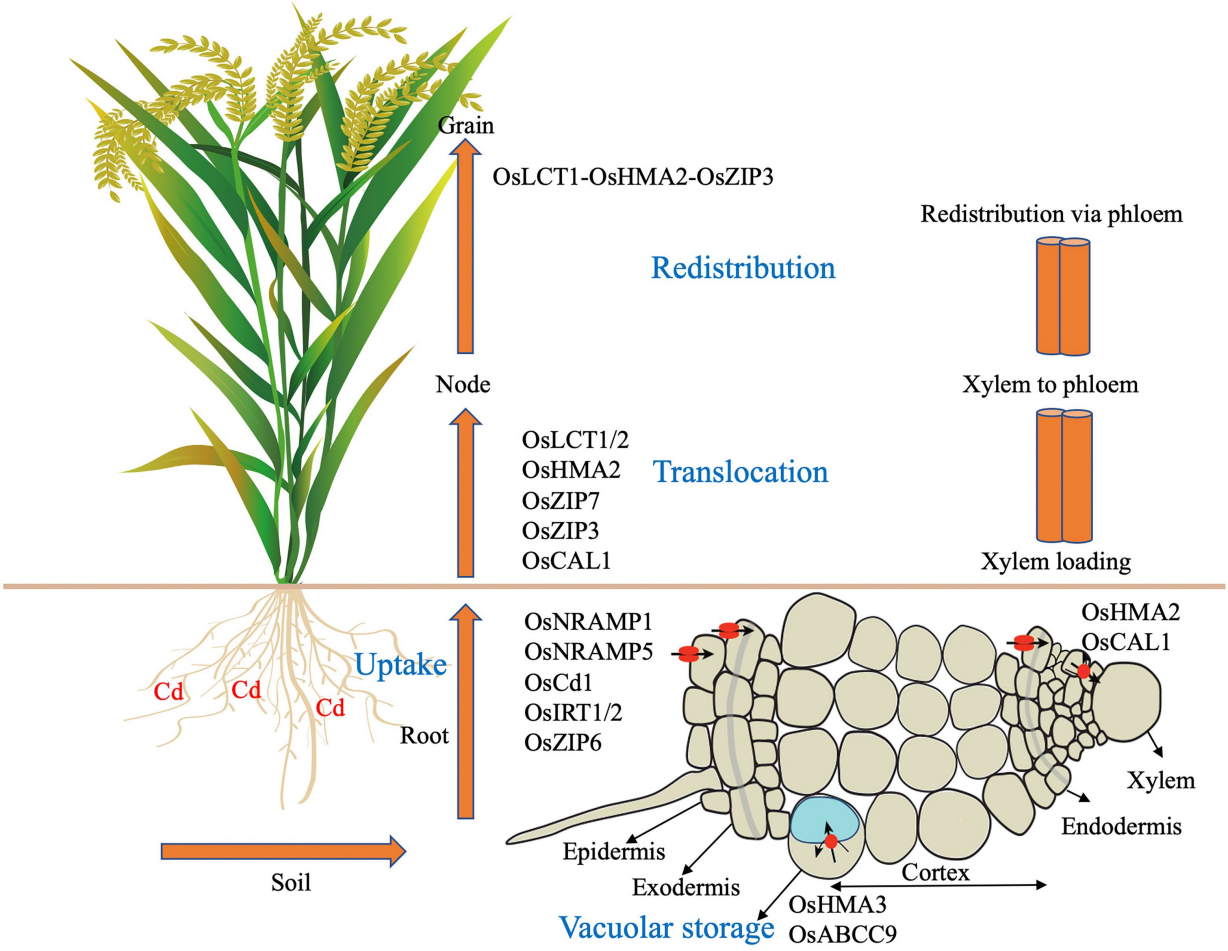
Summary of the contribution of genes function in Cd homeostasis. Cd accumulation in plants is first through root absorption, storage in root vacuoles, xylem loading, xylem to phloem transfer and redistribution. Cd is uptake by rice root in soil. OsNramp1, OsNramp5, OsCd1, and OsITR1/2 are suggested to mediate this process. OsHMA3 and OsABCB9 are response for Cd sequestration into vacuoles in root. OsHMA2 and OsCAL1 contributes to the loading process of Cd into xylem in root. OsLCT1/2, OsHMA2, OsZIP3/7 and OsCAL1 are responsible for intervascular Cd transfer at nodes. Co-expression of OsLCT1-OsHMA2-OsZIP3 could reduce the redistribution of Cd in grains.

The process of Cd absorption and accumulation by plants can be divided into the following steps: absorption of Cd by roots, loading, and transport of Cd into the xylem, transport of Cd from the xylem to the phloem, redistribution of Cd between aboveground stems and leaves, and the accumulation of Cd in grains (Uraguchi et al., 2009). The specific process is shown in Figure 1. Cd in the soil environment passes through layers of obstacles and finally reaches the root xylem, where it is loaded and subsequently transported and unloaded by the xylem and transported longitudinally to different tissues of plant stems, leaves, flowers, and other organs. In addition, after Cd is transported to the aboveground part of the plant, the transport from the xylem to the phloem is completed *via* the dispersed vascular bundle at the stem node (Tanakak et al., 2007).

Higher plants can absorb Cd in soil and water through roots, depending on its availability and concentration in the external medium. In addition, a small part of Cd can be absorbed directly from the atmosphere (Clemens, 2006a). Several factors, such as soil type, pH, climate (temperature, water), the composition of plant rhizosphere microbial community, and genotypic differences of plants, can influence the availability of Cd and plant absorption and transport of Cd (Eriksson, 1989). For example, in Cd-contaminated soils, plant Cd levels were higher at pH 4.0 than at pH 5.0. Soils in several parts of China have become more acidic (especially in the south), such that the activity of Cd in soil has increased and it is easily absorbed by crops (Zhao et al., 2015). The concentration of organic acids in the rhizosphere exerts a great effect on the accumulation of Cd (Lux et al., 2011). The rhizosphere is a small soil area directly affected by root activities; it is affected by root exudates and soil microbial activities. Root exudates play an important role in the bioavailability and toxicity of Cd through controlling rhizosphere pH, redox potential, amount and activity of rhizosphere microorganisms, and chelating ability to Cd. In addition, low molecular weight organic acids secreted by plant roots play an important role in Cd solubility and availability and may mediate the uptake and transport of Cd by plants (Eriksson, 1989). In sandy soils, plants can absorb higher levels of Cd than in clayey soil (Mench and Martin, 1991; Mann and Ritchie, 1995), since most of Cd in sandy soil tend to stay in soluble or exchangeable form (Mann and Ritchie, 1995).

## Cd detoxification mechanism and related genes of Cd absorption and transport in rice

Plants have developed adaptive mechanisms to cope with heavy metal stress, including regulating the absorption of heavy metal ions (Clemens et al., 2002), detoxifying heavy metals through chelation (Clemens, 2006b), and intracellular sequestration (Florijn and Beusichem, 1993) to minimize the exposure to non-essential metal ions (Wang et al., 2011). Plant defense mechanisms against Cd toxicity mainly involve scavenging Cd from active tissues and sequestering it into inactive tissue cells (Song Y. et al., 2017). It is reported that 98% of total Cd remains in *Phaseolus vulgaris* roots, and only 2% of it is transferred to the shoots, and most of the Cd in the roots is located in the apoplast or vacuole (Ouariti et al., 1997). In *Pteris vittata*, Cd is largely distributed in less biologically active tissues such as trichomes and scales (Balestri et al., 2014). However, the uptake and accumulation of Cd by rice was significantly different among different rice varieties (Yu et al., 2006; Ye et al., 2012; Song E. et al., 2015; Luo et al., 2018), for instance, Cd content in *indica* polished grain was higher than that in *japonica* and hybrid grain (Ye et al., 2012). The response of plants to Cd stress is a complex physiological process. Cd enters the plants through the absorption pathway of essential elements (Clemens, 2006a). Studies in yeast, *Arabidopsis*, and rice have revealed that the ATP binding cassette (ABC) family, heavy metal transporting P-type ATPase (HMA) family, ZRT and IRT-like protein (ZIP) family, and natural resistance-associated macrophage proteins (NRAMP) family are involved in the response to Cd homeostasis.

### ATP-binding cassette

ABC transporters are one of the largest families of plants (Tanakak et al., 2007; Uraguchi et al., 2009) and are present in all organisms. These depend on ATP hydrolysis to provide energy and can transfer substances into or out of cells. In plants, ABC transporters are first considered to transport heavy metals and other exogenous substances only on the vacuolar membrane and participate in plant detoxification (Martinoia et al., 1993). Later studies found that they have a wide range of biological functions, such as plant disease resistance, membrane lipid transport, plant inositol formation, and plant hormone transport. Therefore, they play an important role in organ development, plant nutrition uptake, stress resistance, and interaction with the environment (Tanakak et al., 2007). There are 132 members in the ABC family in rice (Garcia et al., 2004; Verrier et al., 2008). Transcriptome studies have demonstrated that several members are involved in the response process of Cd stress and are induced by it; however, its mechanism remains unclear. Among them, *OsABCC9* contributes to Cd vacuolar sequestration in rice roots (Yang et al., 2021). OsABCC9 is located in the tonoplasts of the parenchyma cells, and Cd induces the expression of *OsABCC9*. Knockout of *OsABCC9* resulted in increased sensitivity of rice plants to Cd, these plants accumulated more Cd in roots and shoots, with increased concentration of Cd in the xylem sap and grain, indicating that more Cd is distributed from roots to shoots and grains in *OsABCC9* knocked out plants. *OsABCG43/OsPDR5* and *OsABCG36/OsPDR9* possess Cd transport capacity; which are mainly expressed in rice roots (Moons, 2003; Oda et al., 2011). Heterologous expression of *OsABCG43* in yeast can improve the resistance of yeast to Cd (Oda et al., 2011). In addition, short-time Cd treatment can significantly increase the expression of *OsABCG36* in the roots (Fu et al., 2011). OsABCG36 is located in the plasma membrane (PM). Knocking out *OsABCG36* leads to the accumulation of Cd in the roots and increases the sensitivity of roots to Cd. The expression of *OsABCG36* in yeast shows that it has the efflux activity of Cd (Fu et al., 2011). This indicates that various ABC family genes perform different functions in uptake, transport and distribution of Cd. The genes involved in Cd uptake and transport are summarized in Table 1.

**Table 1:** Genes involved in Cd uptake and transport in rice.

Genes	Location	Function	References
*OsCAL1*	Cell wall	Chelation Cd efflux from the cytosol into extracellular spaces	Luo et al. (2018)
*OsCAL2*	Cell wall	Regulate Cd accumulation	Luo et al. (2020)
*OsABCC9*	Tonoplast	Sequestration of Cd in root vacuoles	Yang et al. (2021)
*OsABCG43/PDR5*	Unknow	Conferring Cd tolerance in yeast	Oda et al. (2011)
*OsHMA2/OsHMA2v*	PM	Transport Cd and Zn from root to shoot	Takahashi et al. (2012), Tian et al. (2019)
*OsHMA3*	Tonoplast	Sequestration of Cd in root vacuoles	Miyadate et al. (2011), Sasaki et al. (2014), Lu et al. (2019)
*OsNRAMP1*	PM	Root uptake and transport of Cd and Mn	Takahashi et al. (2011), Chang et al. (2020)
*OsNRAMP5*	PM	Uptake Mn, Cd and Fe, and transport of these ions from the root to the shoot	Ishikawa et al. (2012), Ishimaru et al. (2012)
*OsLCT1*	PM	Regulate Cd transport into grain	Uraguchi et al. (2011), Tian et al. (2019)
*OsLCT2*	PM	Limiting Cd xylem loading and restricting Cd translocation from roots to shoots	Tang et al. (2021)
*OsZIP1*	PM and ER	Uptake Zn in normal condition, efflux of Zn, Cu and Cd when these metals are excess in environment	Ramesh et al. (2003), Ramegowda et al. (2013), Liu et al. (2019)
*OsZIP3*	PM	Unloading Zn from xylem, Co-expression with *OsLCT1-OsHMA2* could reduce the transport and accumulation of Cd to grains	Sasaki et al. (2015), Tian et al. (2019)
*OsZIP5*	PM	Uptake Cd and Zn	Tan et al. (2020)
*OsZIP7*	PM	Xylem loading of Cd and Zn, distribution of Cd and Zn to grains	Tan et al. (2019)
*OsZIP9*	PM	Uptake Cd and Zn	Huang et al. (2020), Tan et al. (2020), Yang et al. (2020)
*OsIRT1/2*	PM	Uptake Cd, Zn and Fe, translocation of Cd, Zn and Fe to shoot	Nakanishi et al. (2006), Ishimaru et al. (2007), Lee and An (2009)
*OsCd1*	PM	Uptake Cd in root, distribution of Cd into grain	Yan et al. (2019)
*OsCADT1*	Nucleus	Negative regulation of sulfate/selenate uptake and assimilation, increased Cd tolerance	Chen et al. (2020)
*OsO3L2/3*	Nucleus	Reduced Cd accumulation	Wang et al. (2019)
*OsPCR1*	PM	Distribution of Cd and Mn into grains	Song Y. et al. (2015)
*LCD*	Cytoplasm, nucleus	Transport and distribution of Cd into grain	Shimo et al. (2011)
*OsMTP1/OZT1*	Tonoplast	Transport Cd and Zn	Lan et al. (2013), Yuan et al. (2012)
*OsHsfA4a/OsHsfA4b/OsHSF9*	Unknow	Increased Cd tolerance	Shim et al. (2009)
*OsPCS1*	Unknow	Distribution of Cd and As into grains	Das et al. (2017)
*OsCLT1*	Envelope membrane of plastids	Efflux of γ-glutamylcysteine and glutathione from plastids to the cytoplasm, affects As and Cd detoxification	Yang et al. (2016)
*OsHSP18.0-CI/OsMSR3/OsSHSP1*	Cytosol	Enhanced Cd tolerance	Ham et al. (2013), Cui et al. (2019)
*OsCCX2*	PM	Efflux of Cd and loading Cd into xylem	Yadav et al. (2015), Hao et al. (2018)

### HMA family

Heavy metal transporting P-type ATPase is a subfamily of P-type ATPase. The subfamily has relatively conserved domains, including eight transmembrane domains, one CPx domain involved in the transport, and one C-terminal metal ion-binding domain (Colangelo and Guerinot, 2006). Rice genome encodes nine HMA transporters. *OsHMA1-OsHMA3* are transporters of Zn/Cd/Pb/Co divalent cation, whereas *OsHMA4-OsHMA9* belong to Cu/Ag monovalent cation transporters (Williams and Mills, 2005). OsHMA2 is located in the plasma membrane and loads Cd and Zn into the xylem and participates in the transport of Cd and Zn from the roots to shoots. The content of Cd in the seeds of *OsHMA2*-overexpressed lines and *OsSUT1* promoter driving *OsHMA2* transgenic lines is half that of the wild-type; however, the content of other metals is the same as that of the wild-type (Satoh-Nagasawa et al., 2012; Takahashi et al., 2012; Yamaji et al., 2013). Knockout of *OsHMA2* reduced the levels of Cd and Zn in the reproductive tissues of rice (Satoh-Nagasawa et al., 2012; Takahashi et al., 2012; Yamaji et al., 2013). *AtHMA3* has been reported to play a function in the transport of Cd, Zn, Co, and Pb (Morel et al., 2009), whereas *NcHMA3* (*Noccaea caerulescens*) can transport Cd and Zn (Ueno et al., 2011). In rice, *OsHMA3* is mainly expressed in the roots and does not respond to Cd exposure (Ueno et al., 2010). OsHMA3 is located in the tonoplast, and its silencing leads to increased Cd translocation from roots to shoots, whereas its overexpression produces the opposite effect (Ueno et al., 2010; Sasaki et al., 2014). These results suggest that OsHMA3 functions in the transport of Cd into vacuoles and Cd sequestration in the roots, thereby reducing the transport of Cd to shoots (Ueno et al., 2010; Miyadate et al., 2011; Sasaki et al., 2014). Overexpression of *OsHMA3* can also significantly reduce the content of Cd in rice grains. The C-terminal region of OsHMA3, especially the first 105 amino acids, plays an important role in OsHMA3 activity during Cd stress (Kumagai et al., 2014). OsHMA9 has Cu transport activity and can transport Cu outside the cells, which plays an important role in the stability of intracellular concentration of Cu. The expression of *OsHMA9* was induced by Zn and Cd. *OsHMA9* knockout plants were reported to be sensitive to Cd, Zn, Cu, and Pb, and showed a high accumulation of these elements (Lee et al., 2007).

### NRAMP family

Natural resistance-associated macrophage proteins widely exist in microorganisms, plants, and animals, and play an important role in maintaining the dynamic balance of metal ions in organisms. Most of NRAMPs can transport a variety of metal ions, such as Cu, Co, Ni, Cd, Fe, Mn, and Zn (Colangelo and Guerinot, 2006; Nevo and Nelson, 2006; Xia et al., 2010). The rice genome contains seven *NRAMP* genes. *OsNRAMP1* is mainly expressed in the roots and its expression is highly induced by Fe deficiency. OsNRAMP1 is located in the plasma membrane and functions in the transport of Cd and Fe. The difference in Cd accumulation observed in rice varieties is caused by the varied expression of *OsNRAMP1* in the roots (Takahashi et al., 2011). *OsNRAMP5* is an important transporter responsible for Mn uptake by roots, which is necessary for high Mn accumulation in the rice bud. In addition, OsNRAMP5 plays a key role in mediating the entry of Cd into root cells from the outside medium (Sasaki et al., 2012). *OsNRAMP5* is primarily expressed in the exodermis and endodermis of basal root zones, and OsNRAMP5 is located in the plasma membrane in both onion cells and rice protoplasts (Sasaki et al., 2012). The absorption of Cd was greatly reduced in the roots of *osnramp5* mutant, thus reducing the accumulation of Cd in the stems and grains (Ishikawa et al., 2012). Moreover, the *osnramp5* mutant was found to be more sensitive to Mn and Fe deficiencies (Yang et al., 2014). *OsNRAMP2* has two haplotypes owing to four amino acid differences (*OsNRAMP2-L* and *OsNRAMP2-H*) that result in low and high Cd accumulation in rice accessions (Zhao et al., 2018). Using the yeast heterologous assay, the study showed that *OsNRAMP2-L* is the functional form of *OsNRAMP2*; *OsNRAMP2-L* could increase the sensitivity and accumulation of Cd in yeast, whereas *OsNRAMP2-H* could not. However, these four amino acid differences do not affect the localization of distinct haplotype of OsNRAMP2, both OsNRAMP2-L and OsNRAMP2-H were located in the tonoplast, which is different from the localization of OsNRAMP1 and OsNRAMP5 to the plasma membrane (Takahashi et al., 2011; Sasaki et al., 2012).

### ZIP family

The ZIP family is named for its similarity sequence to ZRT1 (Zn-regulated transporter 1) in yeast and IRT1 (Iron-regulated transporter 1) in *Arabidopsis*. It plays an important role in the uptake of metals and is found in several organisms (Connolly et al., 2002). In plants, the ZIP family has been identified both in dicots and monocots (Zheng et al., 2018), such as rice (Chen et al., 2008), *Arabidopsis* (Milner et al., 2013), maize (Li et al., 2013), *Medicago* (Stephens et al., 2011), and *barley* (Tiong et al., 2015). The rice genome consists of 18 ZIP genes (Milner et al., 2013). In rice, *OsITR1* is primarily expressed in rice roots and its expression is induced by Fe deficiency (Bughio et al., 2002). Under Fe deficiency, rice plants tend to accumulate more Cd in roots, indicating that Cd absorption is activated by Fe deficiency. In addition, heterologous expression of *OsIRT1* and *OsIRT2* in yeast increases the sensitivity of Cd and its accumulation in yeast cells (Nakanishi et al., 2006; Lee and An, 2009). *OsZIP1* is considered to be a Zn uptake transporter and induced by Zn deficiency (Ramesh et al., 2003; Bashir et al., 2012; Ramegowda et al., 2013). Further study showed that *OsZIP1* is a metal detoxification transporter that prevents excessive accumulation of Zn, Cu, and Cd in rice (Liu et al., 2019). The *OsZIP1*-overexpression lines grew better and accumulated fewer metals. In contrast, *oszip1* mutants and RNA interference (RNAi) lines accumulated more metals in the roots (Liu et al., 2019). *OsZIP7* loads Zn and Cd into the xylem in rice roots and participates in the intervascular transfer in the nodes by cooperating with OsZIP3 and OsHMA2 (Yamaji et al., 2013; Sasaki et al., 2015; Tian et al., 2019). In addition, co-expression of *OsLCT1-OsHMA2-OsZIP3* can effectively reduce the transport and accumulation of Cd in grains and oxidative stress caused by Cd and Zn stress (Tian et al., 2019). *OsZIP5* and *OsZIP9* are highly expressed in roots and weakly expressed in shoots. OsZIP5 and OsZIP9 function redundantly in Zn/Cd uptake and translocation (Tan et al., 2020).

### Other genes related to Cd tolerance and accumulation in rice

Besides the above ion transporter families, other ion transporters are involved in the absorption and translocation of Cd in rice. For example, a low-affinity cation transporter (LCT) was first found in wheat; *TaLCT1* can transport Ca and Cd and its heterologous expression in yeast leads to increased Cd content in yeast cells, increasing the sensitivity of yeast cells to Cd stress (Clemens et al., 1998). OsLCT1 is located in the plasma membrane of internodal phloem parenchyma cells in rice and promotes the loading of Cd into the phloem sieve tube. Knockout of *OsLCT1* reduced the concentration of Cd in rice grains (Uraguchi et al., 2011). An important role of microRNA (miRNA) has been reported in Cd tolerance in rice (Ding et al., 2018). Reduced expression of *miR166* was found in rice roots under Cd exposure. Overexpression of *miR166* increased Cd tolerance and reduced its transport from roots to shoots, thereby reducing the accumulation of Cd in grains. *OsHB4*, a target gene of miR166, was induced by Cd treatment and downregulated by the overexpression of miR166 in transgenic rice plants. Plants overexpressing *OsHB4* showed increased Cd sensitivity and Cd accumulation in leaves. Conversely, silencing of *OsHB4* enhanced Cd tolerance in transgenic plants (Ding et al., 2018). A putative serine hydroxymethyl transferase OsCADT1 is known to be involved in Cd tolerance; it is a negative regulator of sulfate/selenate absorption and assimilation. In *oscadt1* mutants, the expression of sulfate/selenate transport gene *OsSULTR1* increased, resulting in increased absorption of sulfur and selenium, followed by synthesis of more sulfhydryl compounds to increase the tolerance to Cd (Chen et al., 2020). The *lcd* mutants were screened with Cd tolerance; LCD is localized in the cytoplasm and nucleus and is majorly expressed in the vascular tissues of roots and leaves. The content of Cd in grains of the *lcd* mutant was significantly lower than that in the control when the plants were grown in low Cd-contaminated soil (Shimo et al., 2011). Heat shock transcription factor A4a (HsfA4a) of wheat was screened with Cd tolerance in yeast; the homolog gene of *TaHsfA4a* in rice is *OsHsfA4a*, which could also rescue Cd tolerance in yeast. When *TaHsfA4a* was expressed in rice, it increased the Cd tolerance of rice, whereas the tolerance to Cd was decreased when the expression of *OsHsfA4a* was downregulated (Shim et al., 2009). Overexpression of the full-length sequence or truncated sequence of *OsO3L2* or *OsO3L3* can not only reduce Cd absorption in roots and leaves but also significantly reduce Cd accumulation in grains without affecting the content of other metals such as Mg, Fe, Cu, and Zn (Wang et al., 2016, 2019). Furthermore, the auxin influx transporter *OsAUX1* is involved in primary root and root hair elongation in response to Cd stress (Yu et al., 2015). The expression of *OsPCS1* was induced by Cd in roots and seeds (Das et al., 2017). Both the absence of *OsPCS1* and *OsPCS2* transcripts in developing seeds significantly reduced the content of Cd in the grains by 51% (Das et al., 2017). Cd/cation exchange gene *OsCCX2* is primarily expressed in the xylem region of rice nodes, and OsCCX2 is localized on the plasma membrane. Knockout of *OsCCX2* significantly reduced the Cd content in grains; detailed studies showed that knockout of *OsCCX2* reduced the Cd transport from roots to shoots (Hao et al., 2018).

## Genetic variations and QTLs in absorption and distribution of Cd in rice germplasm

Genotypes play an important role in the absorption, transport, and detoxification of Cd in plants. Differences have been known to exist in Cd accumulation in rice germplasm, which lays the foundation for studying the causes of different Cd tolerance (Arao and Ae, 2003; Ueno et al., 2009a; Pinson et al., 2015). In a recent study, Luo et al. used 212 rice accessions to determine Cd content and found that an *indicia* cultivar Tainan1 (TN1) over accumulated Cd in grains and leaves than cultivar Chunjiang06 (CJ06) (Luo et al., 2018). Further study identified a quantitative trait locus (QTL) mediating Cd accumulation in leaves and named it *CAL1* (Cd accumulation in leaf1). *CAL1* is primarily expressed in the roots and leaf sheaths. Cd treatment significantly induced the expression of *CAL1* in NIL (near isogenic line) -TN1 roots. Cross-section analysis showed that the expression of *CAL1* was superior to that in the xylem parenchyma cells and the vascular system of roots. The accumulation of Cd in leaf blades of seedlings and straws of mature plants in NIL-TN1 was higher than that in NIL-CJ06. This result is consistent with the increased Cd concentration observed in the xylem sap of NIL-TN1. However, no significant difference in Cd level was observed in grains between NIL-TN1 and NIL-CJ06. *In vitro* metal-binding assays showed that CAL1 functions in chelating Cd and facilitating Cd efflux from protoplasts. CAL1 binds Cd in the cytosol, secretes Cd from the xylem parenchyma cells into the xylem vessels, and prevents Cd loading into the phloem, explaining why CAL1 specifically affects the accumulation of Cd in leaves and rice straw but not in grains (Luo et al., 2018). The closest homologous gene to *CAL1* in rice is *OsCAL2*, which is mainly expressed in roots and located in the cell wall (Luo et al., 2020). Moreover, *OsCAL2* also showed Cd binding activity, heterologous overexpression of *OsCAL2* increased the accumulation of Cd in *Arabidopsis* shoots, whereas it decreased the concentration of Cd in roots, and overexpression of *OsCAL2* in rice increased the accumulation of Cd in straws and seeds (Luo et al., 2020). The other gene responsible for Cd accumulation in rice grains between *indica* and *japonica* is *OsCd1* (Yan et al., 2019), which belongs to the major facilitator superfamily (MFS). *OsCd1* is mainly expressed in the roots and its expression is not induced by Cd; it is located in the plasma membrane and involved in Cd uptake. When *OsCd1* is expressed in yeast, it increases the sensitivity of yeast cells to Cd treatment compared with control strains. The difference in Cd accumulation in rice grains between *indica* and *japonica* is ascribed to a natural variation in *OsCd1* and a missense mutation in Val449Asp. In a near-isogenic line (NIL) assay, the introgression line of OsCd1*^V449^* exhibited significantly reduced Cd accumulation in grains compared to 9,311 backgrounds (Yan et al., 2019).

The variations in Cd concentration in the xylem sap are closely related to Cd accumulation in rice shoots and grain, indicating the transport of Cd from roots to shoots as a key process to mediate Cd homeostasis (Uraguchi et al., 2009). Non-functional alleles of *OsHMA3* have been reported in certain *indica* cultivars with high Cd accumulation (Ueno et al., 2010, 2011; Miyadate et al., 2011). Another study revealed that one of these alleles represent a loss of *OsHMA3* function, resulting in decreased vacuolar sequestration of Cd in roots and increased Cd translocation to shoots (Ueno et al., 2010; Miyadate et al., 2011). Its heterologous expression in yeast showed that the 80th amino acid residue of OsHMA3 protein in the high Cd cultivar Anjana Dhan was mutated from Arg to His, resulting in a loss of function (Ueno et al., 2010). In addition, a new loss-of-function allele of *OsHMA3* has been found in certain temperate *japonica* rice varieties with the accumulation of high Cd in shoots and grains (Yan et al., 2016). This allele does not exist in any *indica* rice varieties among 533 rice varieties (Chen et al., 2014) and the sequencing group of 950 world rice varieties (Huang et al., 2012). Compared to the functional allele of *OsHMA3* in *Nipponbare*, the new allele has a single SNP in the coding region, resulting in a mutation of the 380th amino acid from Ser to Arg. Heterologous expression in yeast showed that the new allele of *OsHMA3* haplotype (type II) is nonactive in Cd transport, resulting in the same Cd sensitivity and accumulation phenotypes in yeast as the control and the known nonfunctional allele (type VIII) of Anjana Dhan in yeast. Ser to Arg mutation is expected to alter the charge properties of the OsHMA3 protein without affecting the subcellular localization of OsHMA3 (Yan et al., 2016), which is different, as both the functional (type I) and unfunctional (type VIII) OsHMA3 are mislocated in the endoplasmic reticulum (ER) when expressed in yeast (Ueno et al., 2010). It was also reported that the promoter activity of *OsHMA3* differs between 9,311 and PA64s, resulting in differential *OsHMA3* expression and Cd accumulation in shoots and grain (Liu et al., 2020). This implies that we can reduce the accumulation of Cd in grains through combine different haplotypes of *OsHMA3* and its promoter activity.

Quantitative trait locus (QTL) mapping, a powerful tool to study multivariate genes for complex agronomic traits, has been successfully used to identify loci that control Cd accumulation in rice (Hu et al., 2018). Several QTLs for Cd tolerance have been identified through different genetic populations and phenotypic analysis methods. QTL analysis of Cd accumulation using an F2 population from Anjana Dhan and Nipponbare identified a QTL on chromosome 7 with a significant effect on Cd accumulation, explaining 85.6% of the phenotypic variation in Cd concentration in the shoots of the F2 population (Ueno et al., 2009b). Hu et al. identified certain QTLs related to Cd content in brown rice (CCBR) and Cd content in milled rice (CCMR) using a double haploid (DH) population of Zhongjiadao 17 × D50, of which *qCCBR2-1*/*qCCBR2-2* and *qCCBR9-1*/*qCCBR9-2* were found to be responsible for CCBR, whereas *qCCMR5-1*/*qCCMR5-2* were found to be responsible for CCMR in several fields and pot experiments (Hu et al., 2018). Zhao et al. detected 14 Cd accumulation QTLs in rice grains from 312 rice accessions by a genome-wide association study (GWAS) (Zhao et al., 2018), of which 3 QTLs were first identified in this study, and 4 QTLs were the previously cloned genes (*OsNRAMP1*, *OsNRAMP5*, *OsHMA3*, and *LCD*) that mediate Cd accumulation in rice (Miyadate et al., 2011; Shimo et al., 2011; Takahashi et al., 2011; Sasaki et al., 2012). The grain Cd accumulation QTL of *OsCd1* was identified through the GWAS analysis using 127 rice cultivars (Yan et al., 2019). Natural variations in *OsCd1* in the Val449Asp mutation could distinguish the Cd accumulation in rice grains between *indica* and *japonica*. The Cd accumulation in polished rice was analyzed using 338 distinct rice accessions under Cd-contaminated soil, and 35 QTLs were identified through GWAS in a 2-year assay, of which 9 QTLs were co-localized with a previously reported gene; *OsABCB24* was predicted to be a novel QTL of *qCd1-3* (Pan et al., 2020). A total of 119 Cd-mediated growth response (CGR) -QTLs have been discovered recently, of which 55 have been validated by previously described QTLs, and 64 are novel CGR loci. Certain reported genes have been found to function in CGRs (Yu et al., 2021).

## Future perspectives

With industrialization and urbanization, Cd pollution in soil has become a serious health concern. Solving the problem of soil Cd pollution has become a long-term and arduous task. Cultivating new rice varieties with low Cd, high quality, high yield, and high resistance is one of the effective measures to solve soil Cd pollution and ensure national food security. Under Cd stress, plants regulate the absorption, transport, distribution, and sequestration of Cd by mediating the expression of related genes, thereby balancing the toxicity caused by Cd. However, the limited knowledge of functional genes cannot completely explain the biological processes of Cd homeostasis between tissues and organs and the flow between subcellular organelles in rice. First, although a series of progress has been made regarding the mechanism of Cd absorption, transport, and detoxification in rice, certain genes can be used to remediate Cd-contaminated soil and produce grains with less Cd accumulation. However, these genes are rarely used in popularized varieties. Second, studies on the mechanism of genes involved in Cd stress response are still considerably less and are limited to the uptake and transport of Cd in rice. As well as the upstream regulatory networks of functional genes are still scarce. Third, we need to study the methods to balance different functional genes under different Cd stress to harness the maximum benefit. Plants can induce the expression of several genes under Cd stress; currently, the functions of only a few Cd-induced transporters are known. For example, the expression of *AtHMA3* was induced under Cd stress to accelerate its transport Cd to vacuoles (Mendoza-Cózatl et al., 2011), and nitrate transporter *AtNRT1.8* was also induced by Cd to balance the adverse effects of Cd on nitrogen absorption and assimilation and improve the resistance to Cd (Boussama et al., 1999). Fourth, rhizosphere microbes play an important role in plants responding to Cd stress; however, it is still unclear how to use rhizosphere microbes to remediate Cd-contaminated soil or produce less Cd accumulation in rice grains. Fifth, certain already identified functional Cd absorption and transport proteins, such as OsNRAMP5, have no substrate specificity. Knockout of *OsNRAMP5* is also known to affect the uptake and transport of Mn, thereby reducing the production of rice. Next, we need to study how to uncover the structural basis of functional transporters to balance the uptake of beneficial nutrients and harmful Cd. Sixth, variants of rice germplasm can play an important role in Cd stress, in addition to QTL mapping, we need to combine GWAS and high-throughput transcriptomics to identify more Cd stress response genes and elucidate the underlying molecular mechanism. Seventh, some plants that are super Cd accumulation and tolerant should also be studied, and the mechanism can be applied to the study of cadmium tolerance in rice, this provides a useful supplement to better understand the mechanisms of Cd uptake, transport, and detoxification in plants. We believe these findings will provide a basis for exploring the differences in the characteristics of Cd homeostasis in rice and reducing the environmental and food safety risks and phytoremediation.

## Author contributions

HA and MH wrote the first draft of the manuscript and organized the tables and figures. DW and CL reviewed the manuscript. All authors have read and agreed to the published version of the manuscript.

## Funding

This research was financially supported by the Chinese Postdoctoral Science Foundation (2021M693467), the Natural Science Fund of Education Department of Anhui province (KJ2021A0898), the Talent introduction project in Anhui Science and Technology University (NXYJ202101), and the Natural Science Fund of Anhui Science and Technology University (No. 2021zryb16).

## Conflict of interest

The authors declare that the research was conducted in the absence of any commercial or financial relationships that could be construed as a potential conflict of interest.

## Publisher’s note

All claims expressed in this article are solely those of the authors and do not necessarily represent those of their affiliated organizations, or those of the publisher, the editors and the reviewers. Any product that may be evaluated in this article, or claim that may be made by its manufacturer, is not guaranteed or endorsed by the publisher.
